# Evaluating a capacity development intervention in health economics among producers and users of evidence in Nigeria: a case study in Getting Research Into Policy and Practice (GRIPP) in Anambra State

**DOI:** 10.1186/s13561-022-00371-1

**Published:** 2022-04-23

**Authors:** Charles C. Ezenduka, Obinna E. Onwujekwe

**Affiliations:** 1 Health Policy Research Group (HPRG), Department of Pharmacology and Therapeutics, College of Medicine, University of Nigeria Enugu Campus, Enugu, Nigeria; 2 Department of Health Administration and Management, Faculty of Health Sciences and Technology, College of Medicine, University of Nigeria, Enugu Campus, Enugu, Nigeria

**Keywords:** Capacity development, health economics, health policy and practice, research evidence, training workshop; healthcare system

## Abstract

**Background:**

The use of research evidence to inform policy and practice cannot be overemphasized especially in low and middle-income countries (LMICs). To promote the use of research evidence in the provision of health services for enhanced effective control of communicable diseases in developing countries, the World Health Organization (WHO) in collaboration with the Health Policy Research Group (HPRG) commissioned a capacity development workshop in health economics among producers and users of research evidence in the healthcare system of Anambra state, south east Nigeria. This study was aimed to evaluate the impact of the workshop training on selected stakeholders on the use of health economics evidence to inform health policy and practice in the state.

**Methods:**

Participants were purposively selected based either as producers and users of evidence at various levels of healthcare decision making in Anambra state, comprising mostly senior managers and executives from the ministry of health, the academic and health institutions in the state. A two-day capacity development workshop was conducted to train the participants on the use of economic evidence to inform health policy and practice. Pre-post test approach and group exercises were used to assess the knowledge and impact of the training exercises on the participants regarding the use of health economics evidence. Analysis was based on the framework of process-output-outcome-impact approach using the pre-post test and scores technique to assess the impact of the training programme.

**Results:**

Pretest average scores varied from 39.7% to 60.5% while posttest scores varied from 47.6% to 65.7%, showing big differences in individual scores among participants, between the producers and users of evidence both prior to and after the training. The significant differences between the test scores indicated success in increasing the knowledge of participants on the use of health economics evidence. Results corroborated participants’ perceptions that the workshop impacted positively on their ability to apply the knowledge of health economic evidence to inform decision making in their respective practices.

**Conclusion:**

Findings underscored the need for regular upgrade of stakeholders in the health system for enhanced uptake and sustainability of the programme to achieve the desired goal of getting research into policy and practice in the state applicable to other settings.

## Background

It is a common knowledge that the poor control of the huge burden of endemic diseases in LMICs is attributable to non-use of research evidence which is rarely generated, to inform health policy and practice [1–3]. These evidences hardly exist and where available, are not used appropriately to inform decisions [2]. Notable weaknesses in the health systems of these countries include the scarcity of healthcare professionals with relevant knowledge and expertise in infectious disease research to generate evidence for health policy and practice, in addition to poor use of research evidence to inform policy and practice [2]. Consequently, planning and policy in the system are not well developed for effective implementation and success of healthcare programmes and services [4–6]. In recognition of these gaps, which have negatively impacted on the goals of the healthcare programmes, LMICs including Nigeria have over the years come to increasingly accept the need for the generation and use of research evidence to inform healthcare policy and practice decisions [2–6]. Efforts at getting research evidence to inform healthcare decision-making become a common goal for health managers and decision makers to enhance efficiency and effective healthcare delivery [3–7]. However, many policy and decision makers (users of evidence) lack appropriate capacity to undertake this task. Similarly, many researchers (producers of evidence) lack the requisite skills to generate policy-relevant evidence from health system research and also lack the skills on how to ensure that such generated evidence are used for decision making in Nigeria [2, 4]. It therefore becomes necessary that the capacities of both producers and users of evidence are developed to be able to generate useful evidence from health systems research and policy studies and to use such evidence to inform policy and strategic decisions [2, 6]. Consequently, as part of the efforts at promoting the use of research evidence in the provision of health services aimed at enhancing effective control of communicable diseases in developing countries, the WHO in collaboration with HPRG (University of Nigeria Enugu Campus (UNEC) implemented capacity development workshops in health economics among producers and users of research evidence in the healthcare system of Anambra state, southeast Nigeria. The long-term goal of the intervention is to strengthen individual and institutional capacities in the health system to initiate and lead research activities in disease-endemic countries, while developing national and international partnerships [2, 8].

Given the variations and dimensions in the concept and lack of universal consensus in the definition of the terms ‘capacity building’, ‘capacity development’ and ‘capacity enhancement’ [9–12], the term ‘capacity development’ in this paper is used to capture the notion that capacity already exists and development assistance is building on what is already there, in line with the Australian Agency for International Development (AusAID) (2004), which defined capacity development as “.. the process of developing competencies and capabilities in individuals, groups, organisations, sectors or countries which will lead to sustained and self generating performance improvement" [9, 12]. Hence, in this case capacity development reflects the improvement of existing capacities [13]. Similar to this is ‘capacity strengthening’defined as a “*process of individual and institutional development which leads to higher levels of skills and greater ability to perform useful research*” [5, 14].

To evaluate the success of the capacity development workshop on the participants, from the use of research evidence in policy and practice and achieve the goals of the programme, pre-post test and group exercises were conducted to assess the knowledge and capacity of the participants on the generation and use of health economics evidence to inform health policy and practice. The evaluation was therefore aimed to determine the number of participants with new/increased knowledge of health economic evaluation and analysis. On the whole, analysis was carried out to describe the level of knowledge of the participants regarding the use of economic evaluation evidence to inform healthcare decision making and prioritization, as well as to evaluate the impact of the training by looking at the differences between pre- and post test results.

## Methods

### Study Area

Anambra state is the most populous state southeast Nigeria, with an estimated population of over 5.6 million in 2018 based on the 2006 census, with a density of 860 per km^2^ second to Lagos state [15]. It is governed around three senatorial districts with 21 local government areas (LGAs) made up of 235 districts and 330 political wards. The disease burden of Anambra state has shifted substantially from communicable to non-communicable diseases, with implications for rising costs of healthcare. Non-communicable diseases accounted for 54.8% of inpatient services sought by households, and 54.2% of current health expenditures [15]. The leading causes of outpatient visits include diarrheal diseases, malaria, pulmonary infections, and diseases related to poor hygiene, which can largely be prevented through improved hygiene and behaviour change. Anambra state healthcare system is managed by the State Ministry of Health (SMOH), which is led by the Honourable Commissioner for Health who presides over affairs of the ministry while the permanent Secretary functions as the administrative head. The directors of the different thematic areas report directly to the permanent secretary at the state level while at the LGA levels, the Primary Healthcare (PHC) directors oversee the affairs of the primary healthcare level [16]. The health system is operated under the three-tier system comprising primary, secondary and tertiary levels of care spread across both rural and urban areas. The Primary Health Care is now controlled by the Anambra State Primary Health Care Development Agency (ASPHCDA) in line with the Primary Healthcare Under One Roof (PHCUOR) policy, while the secondary level of care is managed by the State Hospital Management board (SHMB). The two tertiary health facilities in the state; Anambra State Teaching Hospital is run by the Anambra state government and the Nnamdi Azikiwe University Teaching Hospital (NAUTH) at Nnewi is controlled by the Federal Governments. As of 2017 there were over 590 public and 1600 private healthcare facilities operating in Anambra state [16].

### Impact evaluation framework and analysis

The impact assessment framework is anchored on the four levels of: process – output – outcome – impact. This is based on the AusAID’s staged approach to assess, plan and monitor capacity development [12] which focuses on capacity development of the individual and their work group. Hence, in this study the most relevant issue was the focus on evaluation of capacity development activities and outputs, and/or evaluation of the training outcomes. The trainings for capacity development were evaluated using pre- and post tests as a part of standard monitoring and evaluation (M&E) procedures [17].

In order to assess the outcome of the training, the mean of percentages of correct pre- and post test answers/scores were compared [18]. This comparison only allows for evaluation at an aggregated level and does not give a qualitative picture of their knowledge before training or the impact of the training on specific knowledge [5, 17]. Comparing participants’ post-test scores to their pre-test scores enables one to determine whether the training was successful in increasing the knowledge of participants [18]. The group exercises were used to assess participants’ ability to demonstrate their health economics decision making skills, reflecting their practice setting in decision making processes. Hence, while the pre-post tests were used to evaluate the impact of the training on the participants’ knowledge of health economics skills based on a defined/minimum level, group exercises were used to assess participants’ ability/comfort in demonstrating the use of the skill in practice.

### Design and implementation of the capacity development workshops

To achieve the objectives of the capacity development, the workshops adopted a number of activities, which included the identification of the gaps and needs of the researchers and policy makers, capacity building activities in economic evaluation and training workshops. Extensive review of literature was carried out, including the synthesis and use of existing information from health economics and health technology assessment (HTA) literature which are relevant to disease control to improve their design and implementation strategies. This formed the basis for the evaluation of the capacities built on the participants.

The capacity development and impact assessment exercises used a mixed approach to determine the effect of the workshop on the participants, having conducted a desk review of various documents related to capacity development. A variety of instruments were used, including structured questionnaires to obtain information on participants’ demographics, background and their perception and assessment of the workshop and output [18] (Details available in base document) [2]

### Sampling for evaluating the intervention

From the target population a sample of 40 participants were selected, comprising 12 producers and 28 users of evidence from the SMOH, teaching hospitals and the universities in the state. Senior management and executive levels as key stakeholders and main decision makers in the healthcare system were selected. Pre- and post-tests were administered on both the producers and users of evidence at both workshop sessions before and after the training sessions*.* The sample size meets the standard requirement for policy related qualitative studies, limited in subject scope (health economics) and more less varied target population (producers and users of research evidence in health economics) and hence qualified for sample size well below 25, [19, 20].

The participants were administered a 15-item test questions before and after the training with the same set of questions concerning the knowledge and principles of health economics and its use in healthcare decision making/priority setting. Group exercises were conducted for the participants to reflect the practice setting of their services. The workshop was conducted in two sets; the first set was facilitated by workshop facilitators from the Health Policy Research Group (HPRG) while the second set was a step-down training that involved the use of peer educators from the first workshop as facilitators to train another set of stakeholders. The HPRG facilitators provided background support for the peer educators during the step-down training.

While the first workshop was conducted jointly for producers and users of evidence, the step-down sessions were conducted differently for the producers and users of evidence in the state. However, the same evaluation techniques were used in each of the workshop sessions using the pre-post test and group work exercises to assess participants’ uptake of the health economics research tools. Formal approval for the workshop was sought and obtained from the state ministries of health.

### Development of topics and course content

The workshop was divided into relevant sections comprising of didactic and practical/group work sessions. In order to achieve the goals of developing health economics skills for informing efficiency and equity in decision making and resource allocation, it was necessary that appropriate topics and content for the two-day training were carefully chosen to ensure participants’ ease of uptake. Participants’ background on related area was also considered. The content was designed to fit the two-day training period. Case studies were presented with participants working in groups on given exercises/assignments. The review process enabled the authors to identify and prioritize the needs of the producers and users of evidence.

The capacity development and assessment were guided by the following factors: Consideration of the information needs of different audiences; Consideration of both the outcomes and the process; Clear and agreed expectations and indicators; Inclusive indicators that focus on both outcomes and progress in areas that can be influenced; Ensuring that the evaluation framework was flexible rather than fixed; Measuring progress and results in measures other than changed performance; Recognizing that outcomes will not be achieved in a short time frame and often not until after the initiative has finished; and being realistic about what the evaluation could achieve and ensuring expectations are realistic.

Consequently, the following topics were chosen: Concepts and principles of health economics; Value based health care decision making; The role of economic evaluation in healthcare decision making; Cost-effectiveness information in healthcare priority setting; Economic evaluation techniques; Steps in economic evaluation; and Case studies and worked examples

*These were culled from Kotvoijs & Fiona 200 7[*9*]*

### Data collection and analysis

Pre- and post test data were collected from the participants, who completed a 15-point assessment questions. Only the participants who completed both pre and post tests were included in the analysis, while those who completed either a pre-test or a post-test were excluded. Results before and after the training were compared for gains in health economics knowledge.

Pre-test/post-test analysis was used to measure participants’ uptake of training during the workshop. The pre-test results reflect participants’ knowledge of health economics skill prior to the training programme while the post-test findings reflect the impact of the workshop training on participants’ knowledge as part of the goals of the capacity development, to increase participants’ knowledge on the use of health economics evidence in health policy and practice. Pre-post data was based on the number of participants who completed both the pre and post tests exercises and were included in the analysis. Consequently, of the 40 participants, only 25 completed the pre and tests and analyzed accordingly.

Data were analyzed separately for producers and users of evidence for gains in health economics knowledge. One-factor t-tests were used to compare the pre-test and post-test scores with the criterion of success set at 50% . A two-tailed paired t-test was used to compare the pre-test and post-test results. Significance was set at p ≤ 0.05.

#### Criteria of success and gain in ability

This is measured based on a pre-determined level of knowledge or expected gain in knowledge of participants in health economics skill. Differences between pre and post test scores and criteria of success indicates participants’ achievement of expected and/or gain in knowledge prior to and after the training exercises. The extent of change or variation from criteria of success is measured at a significant statistical level of *p* ≤ 0.05. Small or large differences represent deviations from expected or gain in knowledge which could either mean/reflect, depending on deviation from zero (+ or – signs), large, little or no differences from knowledge. This in turn is measured by Cohen’s differences to reflect the magnitude of change in knowledge and ability and therefore impact of the training session. Summary of the criteria of analysis is presented in Table 1.

**Table 1:** Guide to Cohen’s score

Cohen's d scores	Effect size	Implication on criteria of success
-100	Verylarge effect size	The class scores on this test fell far short of meeting the criterion of success for this assessment.
-1.2	Large effect size	The class scores on this test fell short of meeting the criterion of success for this assessment.
-0.8	Medium effect size	The class scores on this test fell somewhat short of meeting the criterion of success for this assessment.
-0.5	Small effect size	The class scores on this test approached the criterion of success for this assessment.
-0.2	Tinyeffect size	The class scores on this test appear to have met the criterion of success for this assessment.
0.2	Smalleffect size	The class scores on this test slightly exceeded the criterion of success for this assessment.
0.5	Mediumeffect size	The class scores on this test measurably exceeded the criterion of success for this assessment.
0.8	Largeeffect size	The class scores on this test greatly exceeded the criterion of success for this assessment.
1.2	Verylarge effect size	The class scores on this test vastly exceeded the criterion of success for this assessment.

Reference: Ferris State University

## Results

### Demographics and summary background of participants

A total of more than 40 participants drawn from Anambra state attended the two-day training workshop, comprising of 40% (16) producers of evidence and 60% (24) users. The proportion of male (48%) participants was close to their female (52%) counterparts.

### Anambra state main workshop

**Fig. 1:**
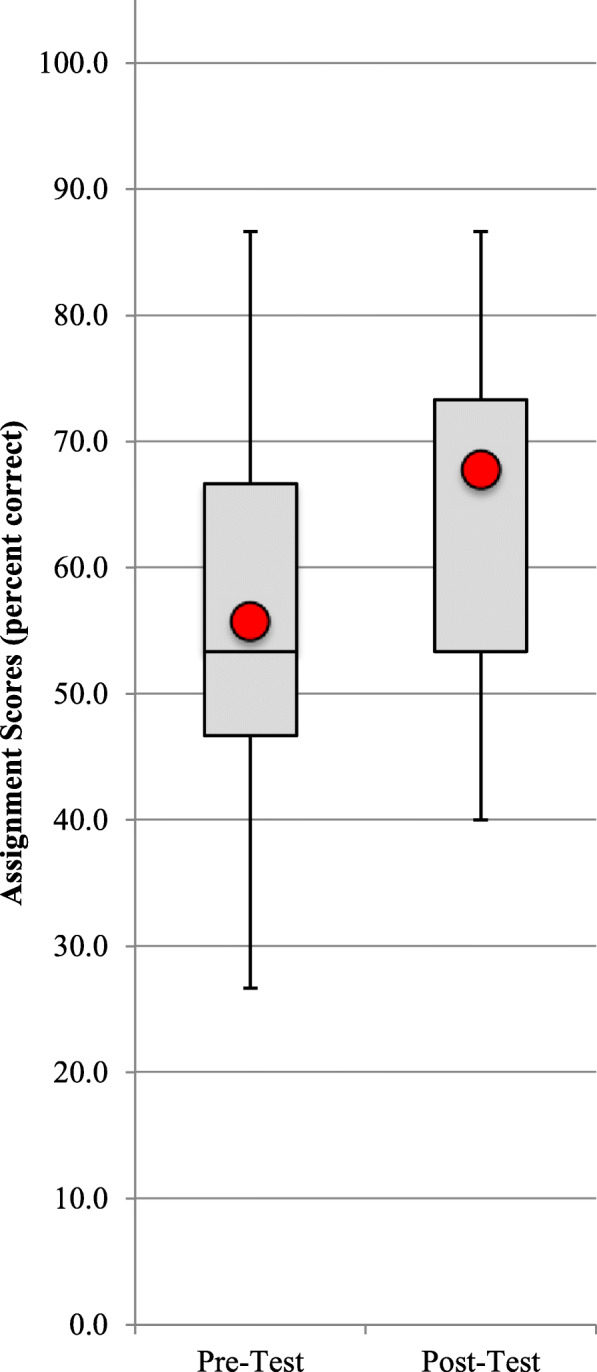
Box and whisker plots of distribution of scores

Table 2 shows that a total of 25 participants, representing both producers and users of evidence completed the pre and post test exercises. The pre-test results recorded a class average of 55.7% ± 5.9% (mean ± 95% CI). A total of 16/25 (92%) students met or exceeded the 50% criterion of success for the pre-test. The difference between the pre-test score and the criterion of success was not statistically significant at t(24) = 1.91, *p* > 0.05. This means that the difference between these scores had a very large effect size (Cohen's d = 1.28), indicating that the pre-test test vastly exceeded the criterion of success for this assessment. The difference between the scores had a small effect size (Cohen's d = 0.38), implying that the class pretest scores slightly exceeded the criterion of success for this assessment. For the post-test, the class scores averaged 67.7% ± 5.4% (mean ± 95% confidence interval). Up to 23/25 (92%) students met or exceeded the criterion of success for the post-test. This time the difference between the post-test score and the criterion of success was statistically significant at t(24) = 6.41, *p* < 0.001. The difference between these scores had a very large effect size (Cohen's d = 1.28), implying it vastly exceeded the criterion of success for this assessment. Fig. 1 box plot illustrates the distribution of the scores between the pre and post tests.

**Table 2:** Summary of Anambra pre-post test results for producers and users of evidence

***Rounded***	***Pre-Test***	***Post-Test***
**N**	25.0	25.0
**Average score (%)**	55.7	67.7
**Std Dev**	15.0	13.8
**Std Err**	3.0	2.8
**95% C.I.**	5.9	5.4
**Min score (%)**	26.7	40.0
**Q1**	46.7	53.3
**Median score (%)**	53.3	73.3
**Q3**	66.7	73.3
**Max score (%)**	86.7	86.7
**Bottom**	46.7	53.3
**Box1**	6.7	20.0
**Box2**	13.3	0.0
**Whisker Dwn**	20.0	13.3
**Whisker Up**	20.0	13.3
**Criterion**	50.0	50.0
**Met**	16.0	23.0
**Percent met**	64.0	92.0
**P**	0.07	0.00
**T**	1.91	6.41
**D**	0.38	1.28
**Comparison**	**Raw**	**Rounded**
Difference	12.00	12.00
s(combined)	14.15	14.10
T	6.19	6.19
P	0.00	< 0.001
D	0.85	0.85
p description		Statistically significant
d description		Large effect size

The results show that the difference between the pre-test and post-test scores (12%) was statistically significant using a two-tailed paired t-test: t(24), *p* < 0.001, in order to determine the change in ability/training impact. The magnitude of this difference has a large effect size (Cohen's d = 0.85), indicating that participants did substantially better on the post-test than they did on the pre-test. Consequently, this suggests a great deal of gain in health economics knowledge over the training workshop.

The gray boxes make up the middle half of all scores (the second and third quartiles) with the median score dividing the two middle quartiles. The whiskers represent the range of the upper and lower 25% of all scores. The red circles represent the average scores.

### Step-down workshops

Table 3 shows the summary findings of the pre-post test for both producers and users of research evidence in Anambra state for the 25 participants that completed pre and post tests for the producers. At pre test, 75% (15/20) of the participants met or exceeded the criteria of success at an average score of 57%. This was not statically significant at t(19) = 1.82, p > 0.05.(Table 3), and at a small Cohen’s effect of d =0.41, the scores slightly exceeded criteria of success. However, at post test, 85% (17/20) of the participants met or exceeded the criteria at average score of about 66%. This was statistically significant at t(19) = 5.38, p < 0.001, leading to a large effect size (Cohen’s d =1.2) which vastly exceeded the criteria of success. Hence, the difference between the pre and post test performance at 8.3%, which was statistically significant on two-tailed paired t-test (t (19), p< 0.025), represents a medium effect size (Cohen’s d = 0.54), indicating a meaningful gain in health economic knowledge by participants.

**Table 3:** Anambra step-down pre-post test results for PRODUCERS and USERS of evidence

	***PRODUCERS***		***USERS***	
***Rounded***	***Pre-Test***	***Post-Test***	***Pre-Test***	***Post-Test***
***Anambra state***				
**N**	20	20	21	21
**Average score (%)**	57.3	65.7	41.3	47.6
**Std Dev**	18.0	13.0	13.3	10.4
**Std Err**	4.0	2.9	2.9	2.3
**95% C.I.**	7.9	5.7	5.7	4.4
***Comparison data***	***Raw***	***Rounded***	***Raw***	***Rounded***
**Difference (%)**	8.33	8.30	6.35	6.30
**s(combined)**	15.32	15.30	11.63	11.60
**T**	2.61	2.61	1.99	1.99
**P**	0.02	< 0.025	0.06	> 0.05
**D**	0.54	0.54	0.55	0.55

For research users, their average pre-test score fell short of the criteria of success, with only 4 of the 21 (19%) participants meeting or exceeded the criteria of success. At Cohen’s d = -0.66, this represents a medium effect that fell short of success. At post-test, while more students 10/21 (47.5%) met the criteria, the difference in scores has a small effect that barely approached the criteria of success. On the whole, the comparative difference in score (6.3%) between the pre and post tests was not statistically significant based on the two-tailed paired t-test (p=>0.05). By Cohen standard, this represents a medium effect size, implying that a better performance was achieved with post-test and a meaningful gain in knowledge of health economics by the Anambra use-participants.

The results show statistical significant differences in the individual scores among the participants, between the producers and users of evidences between the states, both prior to and after the training. In the step-down workshops, the pretest scores ranged from a minimum of 6.7% to a maximum of 86.7% and the posttest from 26.7% to 86.0% for theparticipants. The pretest average scores variedfrom 39.7% to 60.5% while post test scores varied from 47.6% to 65.7%. In all, the lowest pretest scores occurred among the users of evidence.

### Participants’ perception/assessment of course and presentation/responses

The findings showed that most of the participants (82%) rated the course contents as effective. Most of them (74%) felt that the contents were innovative. The survey further revealed that 67% of the respondents felt that the training programs had a positive impact on individual participant given new knowledge and skills imparted, and they had a clear understanding of economic evaluation procedures and operations in healthcare decision making processes. The survey has also revealed that up to 70% of the participants rated the course content very highly. However, majority of the participants felt that the duration of the training was short for the workshop/training content and a little longer duration would have made more impact on participants’ knowledge and capacity.

## Discussion

The result of the two-day training workshops is quite significant. Performances of the participants at both the group work and pre-post tests exercises were significantly positive. It suggests a measured increase in the participants’ knowledge of health economic concepts aimed at improving the efficiency of health policy and practice. As a capacity development training programme the workshop achieved the aim of refining and upgrading the existing skills of the participants in using health economics evidence to inform health policy and practice. Analysis however showed differences in performances and hence impacts between the two sets of trainees and between the producers and users of evidence.

Participants at the initial workshop performed better compared with the step-down participants. This can be attributed to many factors; the fact that information was better communicated by the workshop facilitators who are experts compared with the peer educators who depended on the materials developed by the consultants. The step-down performances by both the producers and users of evidence were lower even though the performances met the criteria of success indicated in all cases. At pre-test, the participants scored above the criteria of success. However, the users in the state showed significant improvement in knowledge during training, scoring within the criteria of success in the post-test, producing larger impact that demonstrated success of the capacity development workshop. In other words, the trainings were successful by increasing participants’ knowledge in the use of health economics evidence.

Overall, the pre-post test results suggest that participants’ knowledge of health economics concept prior to the training was high. This was reflected in the pre test average scores, which in most cases met and exceeded the criteria of success. This occurred for both producers and users of evidence from the state. Exception was however the case for users where only 19% of the participants were able to meet the criteria of success.This was similarly observed in the post test score for the user participants, which although increased to 47%, was unable to meet the criteria of success, even as a measured gain in knowledge was recorded. On the whole, analysis suggests that producers of evidence performed better than their user counterparts. This may be expected given that the majority of producers are mainly researchers from academic environment with related expertise in health economics compared with users that are mainly civil servants.

Notably, participants’ knowledge of economic evaluation principles and the application to inform policy decisions was demonstrated during the group exercises. The performances of the groups showed significant improvement considering the short period of the training. The group work performances and significant increases in the pre-post tests scores demonstrated increase in participants’ knowledge in the use of health economics skills to inform health policy and practice, as a result of the capacity development training programme. Considering the short period of training and diversity of the health economics skills, regular provision of this training and awareness creation will hone the skills of the participants to ensure sustainability and achievement of the long term goals of efficiency in the control of diseases in the healthcare systems.

Participants’ perceptions towards the training programme, in response to the post-evaluation questionnaire, appear to have been reflected in the results of the pre-post test and group exercises, reinforcing the success of the training programme. The majority response that the programme has impacted positively on their knowledge of health economics skill implies that the programme was able to increase their ability to apply the evidence in health decision making processes, a key goal of the workshop. However, some of the responses/perceptions which also reflected in the results of the exercises highlighted many limitations and concerns that need to be considered for improvement in the future to enhance the achievement of the capacity development goals, such as in course content, duration of training, regular training, choice/selection of appropriate/quality peer educators etc. Generally, performance indicated significant increase in the level of participants’ knowledge in health economics as the results for most questions showed significant increases after the training, to a level of around 70% with correct answers. The study in Malawi 2014 which assessed the impact of capacity development among public service administrators reported similar findings even though the training took over three weeks period [21].

It is important to point out that, like in many of the related studies, the pre-test/post-test analyses have no control group, they tend to have lower validities [17, 18, 21]. It becomes necessary to be cautious in making inferences regarding the cause of any changes in the participants’ performances. Given the limited impact of the step-down workshop compared with the outcome of the first workshop facilitated by the HPRG consultants, it is necessary to review the suitability of resource persons used as peer educators in providing the step-down trainings. Otherwise, one workshop with probably larger number of participants facilitated by the HPRG consultants would guarantee better results.

The capacity development workshop has demonstrated increased in participants’ knowledge of health economics principles and its application to inform health policy and practice. The findings also corroborated participants’ perceptions that the workshop impacted positively on their ability to apply the knowledge of health economic evidence to inform decision making in the healthcare system. Considering the short period of training and assessment, findings underscore the need for significant and regular upgrade of participants’ knowledge on the use of health economic evidence to inform decision making in the state’s healthcare system. Similarly the findings that majority of participants have positive disposition towards the training also reflected in the measured impact of the short workshop, indicating the potential for achieving the overall goal of the project if appropriate actions are taken and recommendations implemented.

However, it should be noted that while the sample size of this study may appear constraining, it nonetheless falls within the standard range for policy related qualitative studies depending on the study scope and population [19, 20]. Our study is not only limited to health economics subject but a more homogenous/less varied target population of stakeholders which qualifies for sample size well below 25 [20]. Hence, as a policy and capacity-development related study, the findings provide invaluable insights on the subject for other contexts, even if they are not directly generalizable to the entire country. The findings are also useful and relevant to decision makers in related settings. However, future studies that are well resourced can benefit from the use of more study sites and larger sample sizes for increased generalisability of the findings.

## Conclusion

The paper shows that the bridge between non-use of evidence and use of evidence for decision making can be built with appropriate recognition of capacity gaps and actions to bridge these gaps in the quest for making evidence-informed decisions in the health system. The study findings provide opportunities to scale-up and institutionalize capacity building processes in the state to strengthen capabilities in the health system for making evidence-informed decisions in order to enhance the effectiveness, efficiency and equity of disease control activities in both the state and in similar settings. As a test case the study indeed provide useful insights on the capacity of relevant stakeholders to use scientific evidence to inform policy and practice, in recognition of the poor use of such evidence in LMICs for effective control of diseases. The ultimate goal of individual and institutional capacities for evidence-based research activities is to strengthen the health system to enhance the efficiency and effective control of communicable diseases.

## Data Availability

The relevant datasets used for the current study are available from the corresponding author on request where necessary.

## Abbreviations

GRIPP: Getting Research into Policy and Practice
HPRG: Health Policy Research Group
HTA: Health Technology Assessment
LMICs: Low and Middle-Income Countries;
NCD: Non-communicable diseases
PHC: Primary Health Care
PHCUOR: Primary Health Care Under One Roof
SMOH: State Ministry of Health
UNEC: University of Nigeria, Enugu Campus
WHO: World Health Organization
